# How to risk stratify new‐onset atrial fibrillation in patients with hypertension and diabetes mellitus

**DOI:** 10.1002/joa3.12963

**Published:** 2023-11-27

**Authors:** Naoya Kataoka, Teruhiko Imamura

**Affiliations:** ^1^ Second Department of Internal Medicine University of Toyama Toyama Japan

To editor,

Kikuko and colleagues have elucidated that an arbitrary blood glucose level and the concomitance of valvular heart disease stand as substantial risk factors for the inception of new‐onset atrial fibrillation (NOAF) in hypertensive and diabetic patients.^1^ Numerous concerns, warranting resolution, have arisen with respect to their findings.

Firstly, their study's explication of each prospective risk factor remains opaque.^1^ The precise categorization of valvular heart disease in terms of its severity and type has been conspicuously omitted. In the event that this encompasses mitral stenosis, the novelty of their discovery becomes shrouded in uncertainty. Additionally, the precise nature of the underlying infections is also shrouded in ambiguity. Prior literature has demonstrated a linkage between sepsis and atrial fibrillation development,^2^ thus obscuring the originality of their findings.

It is well‐documented in the scientific domain that various anatomical and functional parameters of the left atrium are established risk factors for NOAF, notably encompassing parameters such as left atrial strain and left atrial volume index. For example, Park and colleagues have illustrated that a novel scoring system, integrating diminished peak atrial longitudinal strain, offers valuable insights into the determination of NOAF risk.^3^ The pertinent query arises: Did the authors incorporate measurements of these left atrial parameters into their investigation?

The accurate identification of NOAF constitutes another salient concern. The authors have merely asserted that NOAF was defined as the initial or newfound diagnosis of atrial fibrillation, irrespective of symptomatic manifestation.^1^ However, it is well‐recognized that a significant proportion of atrial fibrillation cases remain asymptomatic, potentially resulting in the inadvertent omission of numerous NOAF occurrences.^4^ Conversely, transient episodes of high atrial rates, which are discerned in patients with implanted pacemakers, do not mandate anticoagulation therapy for the prevention of cerebrovascular events.^5^ Could the authors offer an elucidation of their methodology for the identification of NOAF, along with insights into the potential risks associated with undiagnosed NOAF?

## CONFLICT OF INTEREST STATEMENT

Authors declare no conflict of interests for this article.
